# Supplementary material for: Imprinting fidelity in mouse iPSCs depends on sex of donor cell and medium formulation
Source: Nat Commun. 2022 Sep 16;13:5432. doi: 10.1038/s41467-022-33013-5 (PMC9481624; doi:10.1038/s41467-022-33013-5)
Supplement: Supplementary file 1 — Supplementary Information [file 41467_2022_33013_MOESM1_ESM.pdf]

**Imprinting fidelity in mouse iPSCs depends on sex of donor cell and medium formulation**

Maria Arez, Melanie Eckersley-Maslin, Tajda Klobučar, João von Gilsa Lopes, Felix Krueger, Annalisa Mupo, Ana Cláudia Raposo, David Oxley, Samantha Mancino, Anne-Valerie Gendrel, Bruno Bernardes de Jesus, Simão Teixeira da Rocha

## SUMMARY OF SUPPLEMENTARY INFORMATION

**Supplementary Figure 1 –** Expression analysis of pluripotent markers in F1 hybrid KSR-iPSCs

**Supplementary Figure 2 –** Widespread imprinting methylation defects in KSR-iPSCs

**Supplementary Figure 3 –** RNAseq data of KSR-iPSCs

**Supplementary Figure 4 –** Generation of F1 hybrid FBS-iPSCs

**Supplementary Figure 5 –** Imprinting defects in the *Dlk1-Dio3* locus in female and male KSR- and FBS-iPSCs

**Supplementary Figure 6 –** Expression analysis of genes involved in the DNA methylation machinery and imprinting protection

**Supplementary Figure 7 –** Imprinting and global 5mC/5hmC dynamics during reprogramming and maintenance of iPSCs

**Supplementary Figure 8 –** Persistence of imprinting errors in iPSC-derived NPCs

**Supplementary Figure 9 –** Expression of imprinted genes in KSR/FBS- and FBS+VitC-iPSCs

**Supplementary Figure 10 –** FACS gating strategy

**Supplementary Table 1 -** Previous studies addressing imprinting defects in mouse iPSCs

**Supplementary Table 2 -** Primers used for pyrosequencing, RT-qPCR, genotyping and allelic expression experiments

**Supplementary Table 3 -** Primers used for IMPLICON experiments

# Supplementary Fig. 1

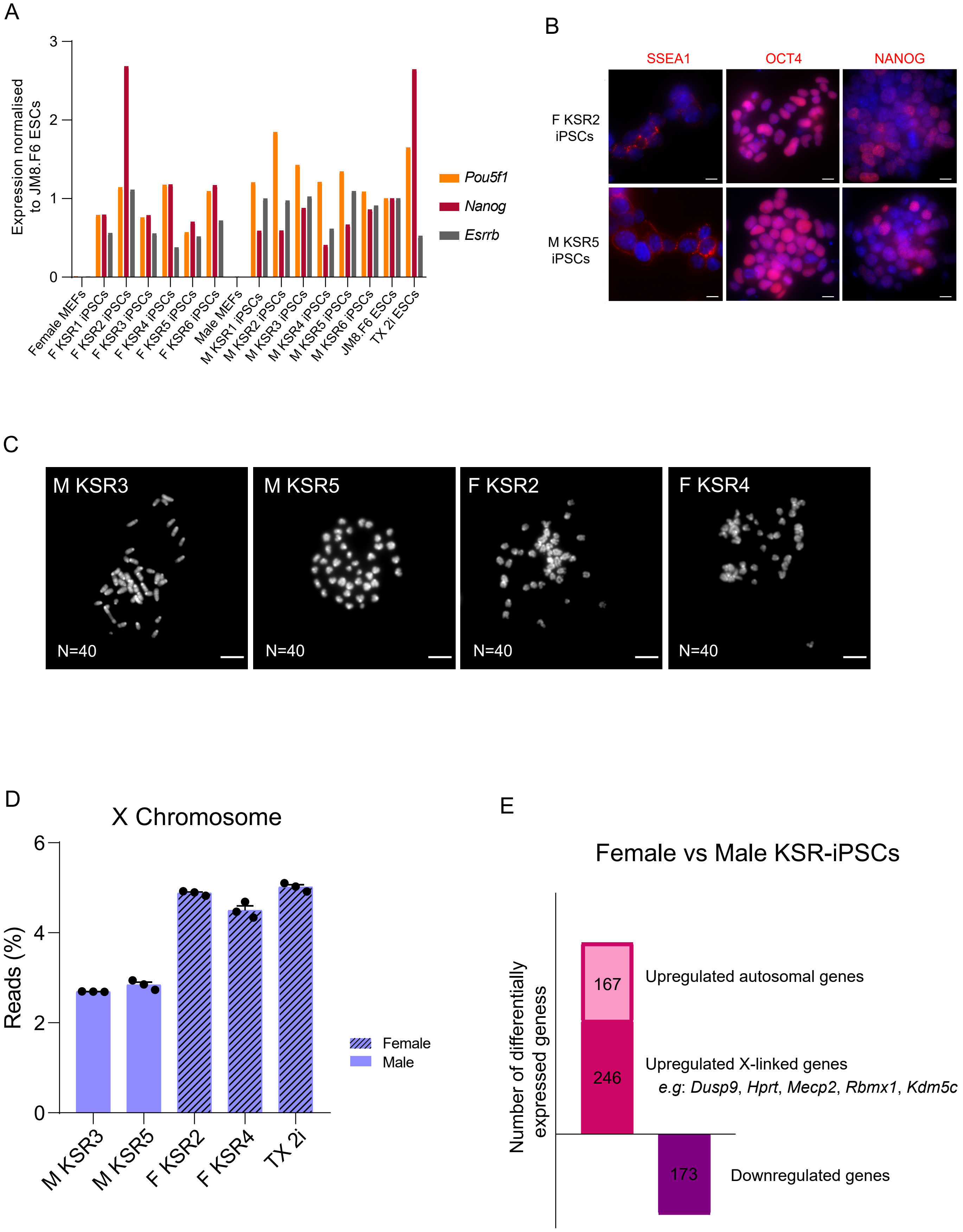

**Supplementary Figure 1 – Expression analysis of pluripotent markers in F1 hybrid KSR-iPSCs**

A. RT-qPCR expression analyses of pluripotent markers (*Pou5f1*, *Nanog* and *Esrrb*) normalised with the *Gapdh* housekeeping gene in female MEFs, female (F KSR1-F KSR6), male (M KSR1-M KSR6) iPSCs and JM8.F6 and TX 2i ESCs; Values for each gene were normalised to the JM8.F6 ESCs; each bar represents data from only one biological replicate. Source data are provided as a Source Data file.

B. Representative immunofluorescence (IF) images for three pluripotent markers (SSEA1, OCT4 and NANOG) in red and nuclei in blue (DAPI staining) in F KSR2 and M KSR5 iPSCs; This experiment was performed once per cell line for each antibody. Scale bars correspond to 10 μm.

C. Representative karyotypes of M KSR3, M KSR5, F KSR2 and F KSR4 iPSC lines with 40 chromosomes each (N=40); Scale bars correspond to 10 μm; A normal karyotype was considered when most of the metaphases counted presented 40 chromosomes (see Methods).

D. Percentage of total RNAseq reads mapping on the X chromosome in biological triplicates of male (M KSR3 and M KSR5), female (F KSR2 and F KSR4) iPSCs and TX 2i ESCs. Graph represents the mean percentage ± SEM of reads mapping on the X chromosome per cell line. Source data are provided as a Source Data file.

E. Differential expression analysis between female and male KSR-iPSCs using both EdgeR and intensity difference filter (*p*-value < 0.05 with multiple testing correction using Benjamini and Hochberg correction for both). Graph represents the number of upregulated and downregulated genes in female versus male KSR-iPSCs. The number of differentially expressed genes from the X chromosome is highlighted. There are no downregulated X-linked genes. Source data are provided as a Supplementary Data 1.

Supplementary Fig. 2

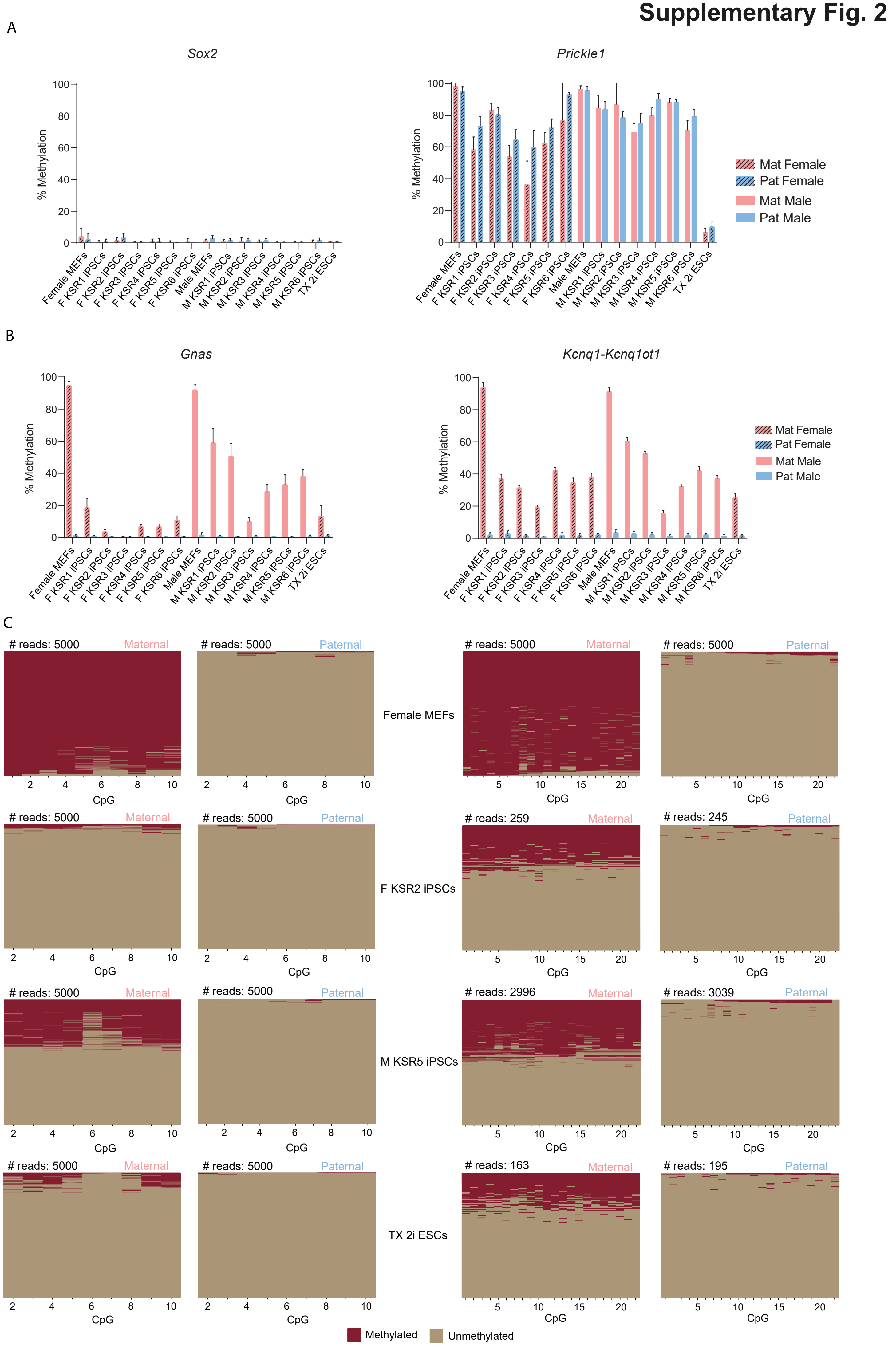

**Supplementary Figure 2 – Widespread imprinting methylation defects in KSR-iPSCs**

A. Methylation analysis of *Sox2* (unmethylated control) and *Prickle1* (methylated control) in male and female MEFs, female (F KSR1-F KSR6) and male (M KSR1-M KSR6) iPSCs and TX 2i ESCs; Each graph represents the mean ± SD methylation levels measured at each CpG within different genomic regions per parental allele for each sample (number of CpG per locus - *Sox2*: n=14; *Prickle1*: n=10); Source data are provided as Supplementary Data 2.

B. Methylation analysis of *Gnas* and *Kcnq1-Kcnq1ot1* imprinted loci in female and male MEFs, female (F KSR1-F KSR6) and male (M KSR1-M KSR6) iPSCs and TX 2i ESCs; Each graph represents the mean ± SD methylation levels measured at each CpG within different genomic regions per parental allele for each sample (number of CpG per locus - *Gnas*: n=10; *Kcnq1-Kcnq1ot1:* n=23); Source data are provided as Supplementary Data 2.

C. Plots display methylated and unmethylated CpGs for each CpG position (in columns) in all the individual reads (in rows) for both maternal and paternal alleles of *Gnas* and *Kcnq1-Kcnq1ot1* imprinted loci in female MEFs, F KSR2 and M KSR5 iPSCs and TX 2i ESCs.

## Supplementary Fig. 3

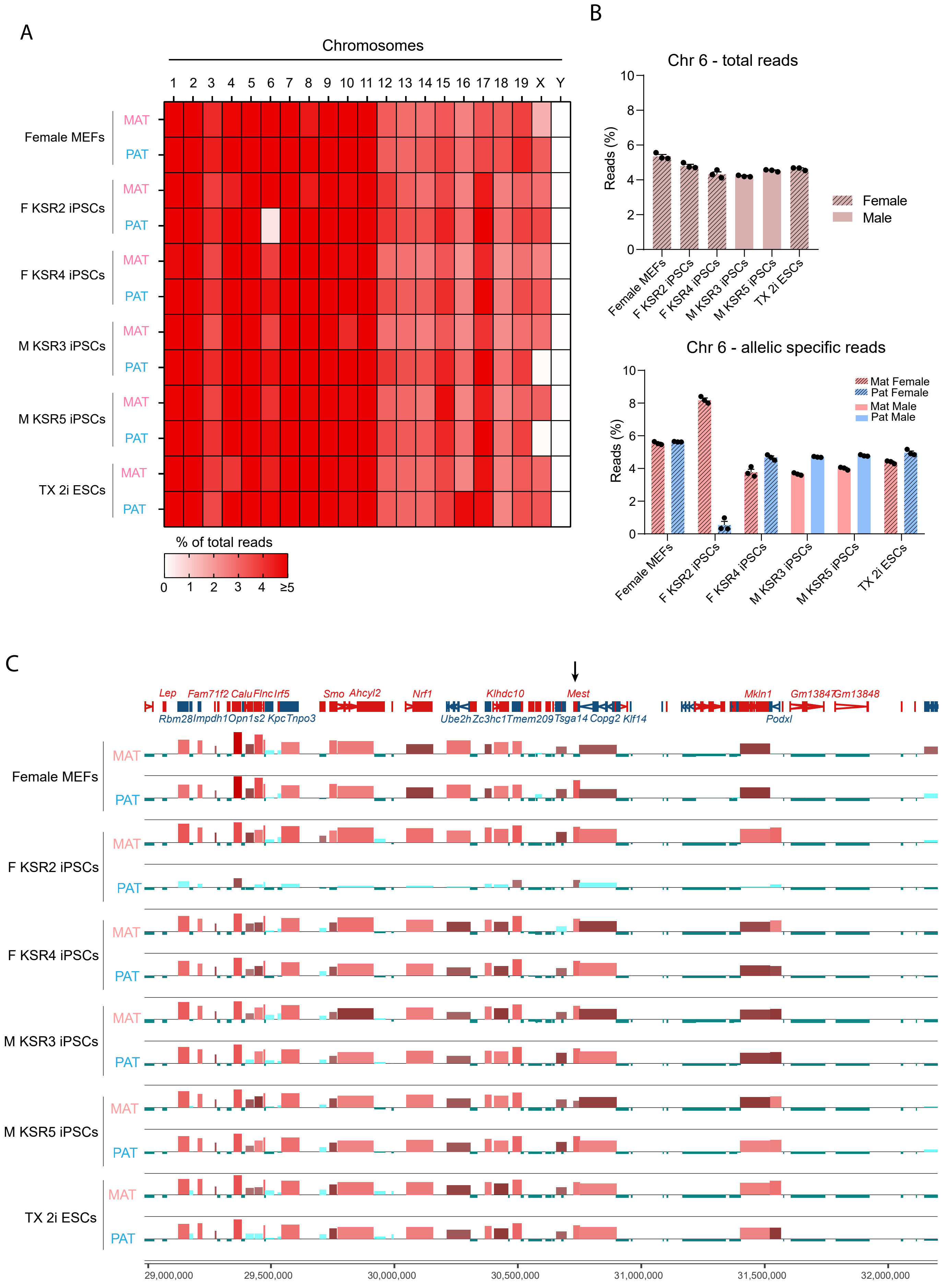

**Supplementary Figure 3 - RNAseq data of KSR-iPSCs**

A. Heatmap representing the percentage of total reads of each parental chromosome (MAT - maternal; PAT - paternal) in biological triplicates of female MEFs, female (F KSR2 and F KSR4) male (M KSR3 and M KSR5) iPSCs and TX 2i ESCs. Note: absence of Y chromosome in M KSR3 and M KSR5 is due to low number of confident SNPs for this chromosome (1132) compared to others (e.g, X chromosome – 636442 SNPs). Source data are provided as a Source Data file.

B. Percentage of total RNAseq reads of chromosome 6 (top) and of allele-specific reads distinguishing the two parental chromosomes 6 (bottom) in biological triplicates of female MEFs, female (F KSR2 and F KSR4), male (M KSR3 and M KSR5) iPSCs and TX 2i ESCs. Graphs represent the mean percentage ± SEM of total (top) and allele-specific (bottom) reads mapping on the chromosome 6 per cell line. Source data are provided as a Source Data file.

C. Genome browser view of chromosome 6 region containing *Mest* imprinted gene (denoted by black arrow); Height of bars correspond to log2 RPKM values for each gene on either maternal (MAT) or paternal (PAT) inherited allele for biological triplicates of female MEFs, female (F KSR2 and F KSR4) male (M KSR3 and M KSR5) iPSCs and TX 2i ESCs. Selected genes names on sense (red) and antisense (dark blue) are shown.

## Supplementary Fig. 4

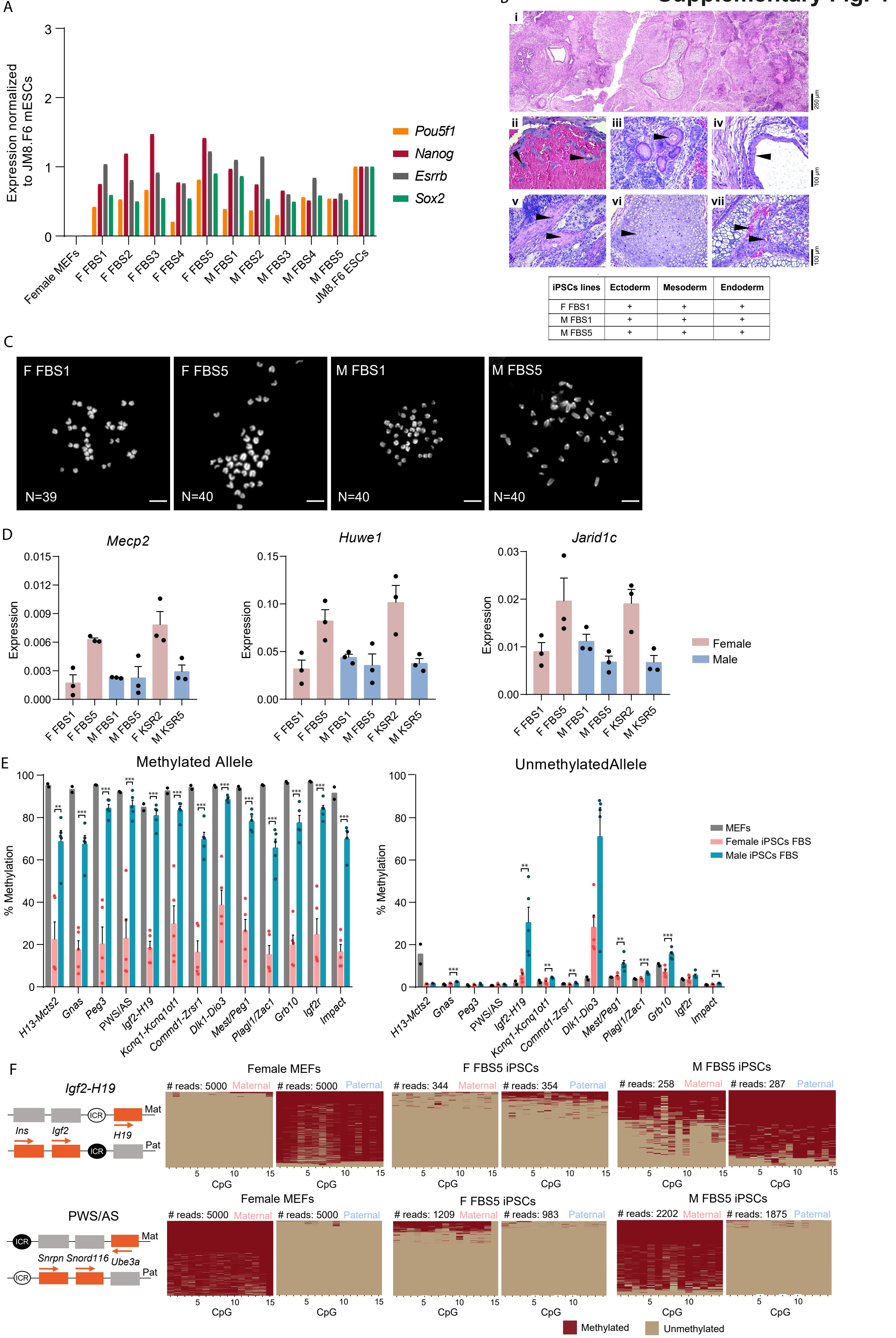


| iPSCs lines | Ectoderm | Mesoderm | Endoderm |
|---|---|---|---|
| F FBS1 | + | + | + |
| M FBS1 | + | + | + |
| M FBS5 | + | + | + |

**Supplementary Figure 4 – Generation of F1 hybrid FBS-iPSCs**

A. RT-qPCR of pluripotent markers (*Pou5f1*, *Nanog*, *Esrrb* and *Sox2*) normalised to the *Gapdh* housekeeping gene in female MEFs, female and male FBS-iPSCs and JM8.F6 ESCs; Values are normalised to JM8.F6 ESCs; Each bar represents data from only one biological replicate. Source data are provided as a Source Data file.

B. Table and representative H&E staining of teratomas derived from FBS-iPSCs; **i**, Low magnification of a mature teratoma. **ii**, Trophectoderm-derived trophoblast giant cells, associated with large vascular spaces (black arrowhead). **iii**, Ectodermal components corresponding to squamous epithelium (black arrowhead). **iv**, Endodermal components corresponding to ciliated respiratory epithelium (black arrowhead). **v, vi, vii**, Mesodermal components (black arrowhead) corresponding to fibrous tissue, cartilage, and blood vessels; Table summarises the successful generation of trilineage teratomas from F FBS1, M FBS1 and M FBS5 iPSCs. Two teratomas per cell line were analysed by H&E staining.

C. Representative karyotypes of F FBS1, F FBS5, M FBS1 and M FBS5 iPSC lines with 39 chromosomes for the F FBS1 and 40 for the others. Scale bars correspond to 10 µm; Number of chromosomes considered per each line corresponds to the most frequent pattern present in the metaphases counted (see Methods);

D. Graph represents mean expression ± SEM measured by RT-qPCR of X-linked genes (*Mecp2*, *Huwe1* and *Jarid1c*) normalised to the *Gapdh* gene in F FBS1, F FBS5, M FBS1, M FBS5, F KSR2 and M KSR5 iPSCs (n=3 biological replicates). Source data are provided as a Source Data file.

E. Graph represents the mean percentage of methylation ± SEM at methylated and unmethylated alleles of ICRs in both parental MEFs (note: same data as in Fig. 2B, n=2 independent cell lines), female and male FBS-iPSCs (n=5 independent cell lines each); Statistically significant differences between female and male

FBS-iPSCs are indicated as ** $p < 0.01$; *** $p < 0.001$ (unpaired two-tailed Student's *t*-test). Source data are provided as Supplementary Data 2.

F. Plots displaying methylated and unmethylated CpGs for each CpG position (columns) in all the individual reads (rows) for both maternal and paternal alleles of *Igf2-H19* and PWS/AS loci in female MEFs, F FBS5 and M FBS5 iPSCs; Schemes on the left represent the normal methylation status in *Igf2-H19* and PWS/AS loci (as in Fig. 2A).

## Supplementary Fig. 5

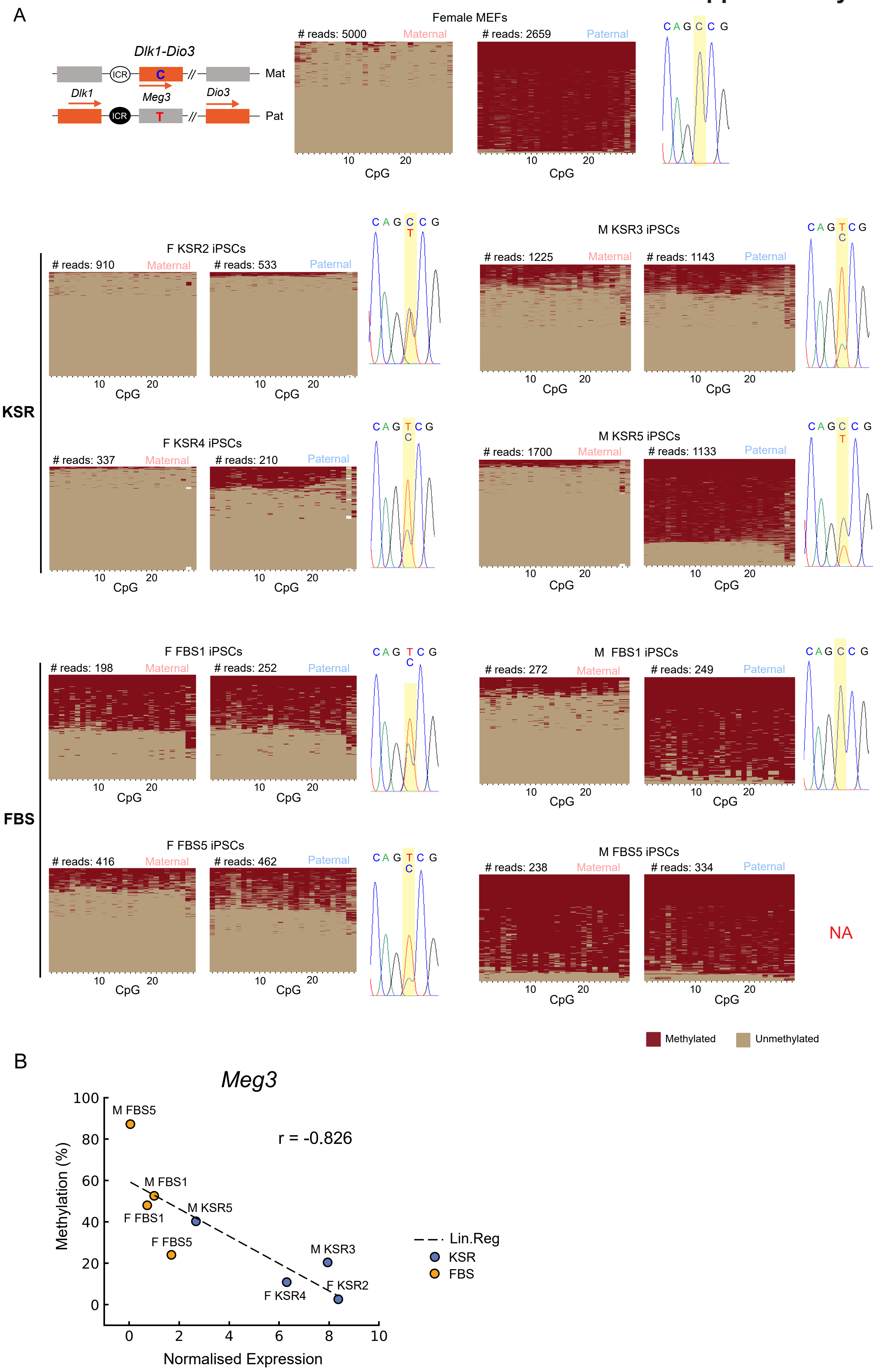

**Supplementary Figure 5 – Imprinting defects in the *Dlk1-Dio3* locus in female and male KSR- and FBS-iPSCs**

A. Plots displaying methylated and unmethylated CpGs for each CpG position (in columns) in all the individual reads (in rows) for both maternal and paternal alleles of *Dlk1-Dio3* locus in female MEFs, female and male KSR-iPSCs (F KSR2, F KSR4, M KSR3, M KSR5) and female and male FBS-iPSCs (F FBS1, F FBS5, M FBS1, M FBS5) with the correspondent chromatograms displaying allelic-specific *Meg3* expression assayed by Sanger sequencing on their right. M FBS5 does not express *Meg3*, hence allelic expression was not performed (NA - not applicable); scheme on the left of female MEFs plot represent the normal imprinting status of *Dlk1-Dio3* locus with normal *Meg3* expression and with the associated SNP (in blue) of each allele; white circle – unmethylated ICR; black circle – methylated ICR; Mat – maternal allele; Pat – paternal allele; orange rectangle – expressed gene; grey rectangle –silenced gene; region is not drawn to scale.

B. Scatter plot representing the correlation between *Dlk1-Dio3* ICR methylation and *Meg3* expression for the samples: F KSR2, F KSR4, M KSR3, M KSR5, F FBS1, F FBS5, M FBS1 and M FBS5. The X-axis represents the average methylation levels of the *Dlk1-Dio3* ICR considering both parental alleles. The Y-axis represents the *Meg3/Gapdh* values measured by RT-qPCR expression analysis and normalised to the M FBS1 iPSC line (n=3 biological independent replicates). *r* represents Pearson's correlation between methylation levels of *Dlk1-Dio3* locus measured by IMPLICON and *Meg3* expression data measured by RT-qPCR. Source data are provided as a Source Data file.

# Supplementary Fig. 6

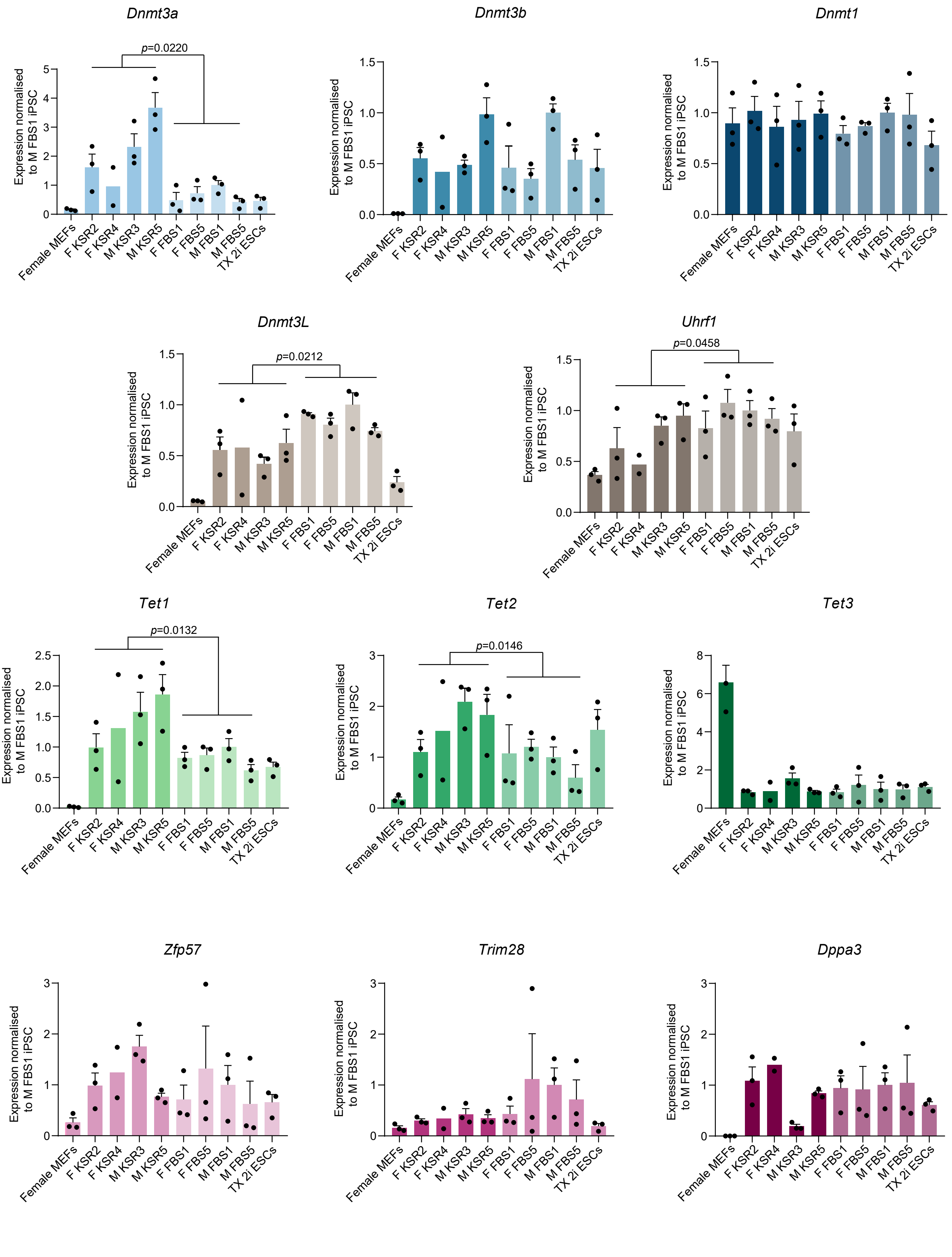

**Supplementary Figure 6 – Expression analysis of genes involved in the DNA methylation machinery and imprinting protection**

RT-qPCR expression analyses for *Dnmt3a*, *Dnmt3b*, *Dnmt1*, *Dnmt3L, Uhrf1, Tet1*, *Tet2*, *Tet3*, *Zfp57*, *Trim28* and *Dppa3* normalised with the *Gapdh* housekeeping gene in female MEFs, F KSR2, F KSR4, M KSR3, M KSR5, F FBS1, F FBS5, M FBS1, M FBS5 iPSCs (n=3 biological independent replicates; except for F KSR4 iPSC, where n=3 only for *Dnmt1* and n=2 biological replicates for all the other genes). Graph represents the mean expression ± SEM for each cell line. Values for each gene were normalised to the M FBS1 iPSC. *P*-values < 0.5 comparing KSR- and FBS-iPSCs are indicated on top of the bars (two-way ANOVA). Source data are provided as a Source Data file.

# Supplementary Fig. 7

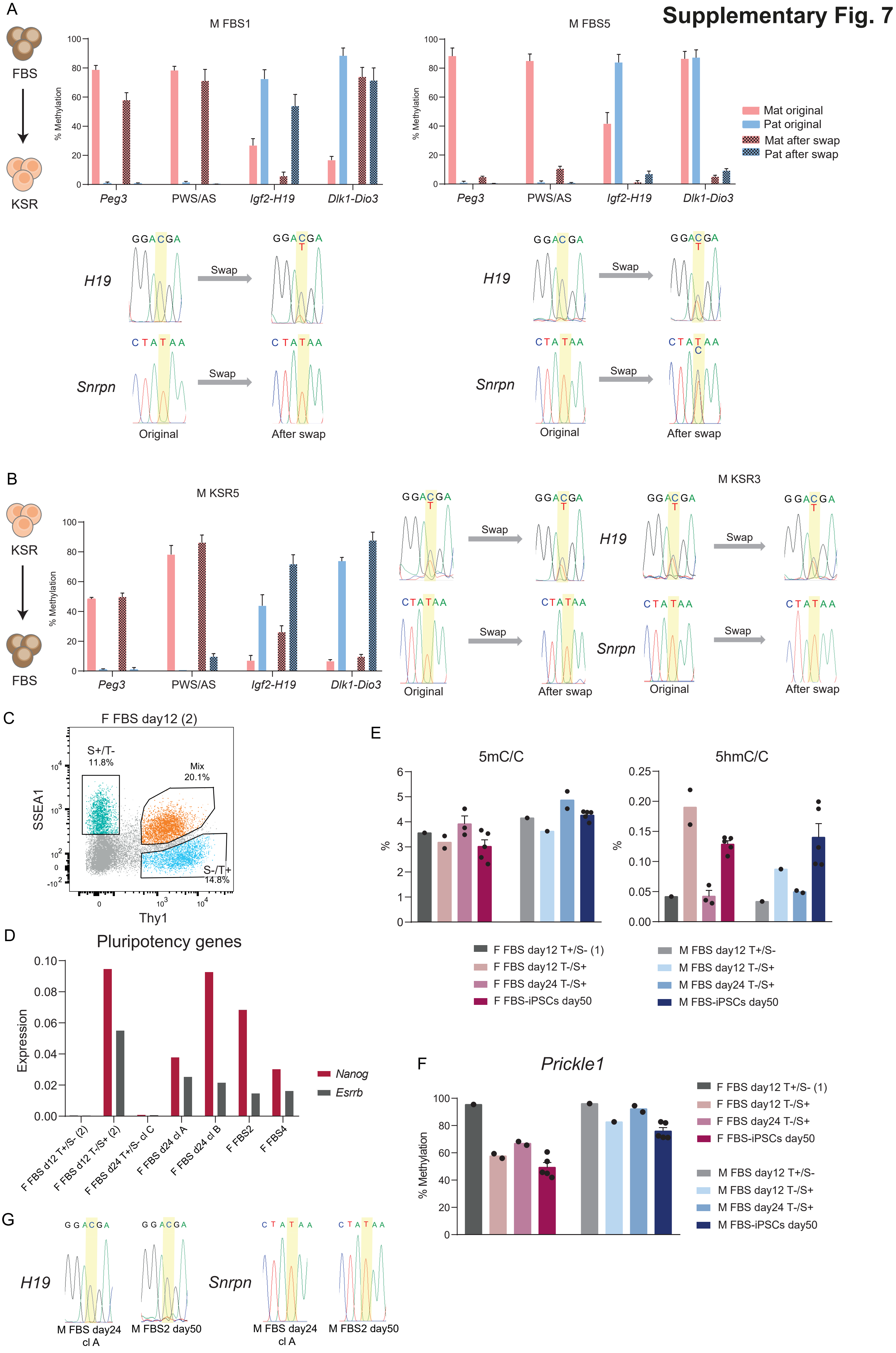

**Supplementary Figure 7 – Imprinting and global 5mC/5hmC dynamics during reprogramming and maintenance of iPSCs**

A-B. FBS-to-KSR (A) and KSR-to-FBS swap (B) experiments – briefly, M FBS1 and M FBS5 previously generated in Foetal Bovine Serum-based medium (FBS) were cultured for 10 passages (~20 days) in Knockout Serum Replacement-based medium (KSR), while M KSR3 and M KSR5 previously generated in KSR medium were cultured in FBS medium. Imprinting methylation was measured after swap by IMPLICON and imprinting expression was measured by Sanger Sequencing. Graphs on top in A and B show mean ± SD methylation levels measured at each CpG within different imprinted regions per parental allele for each sample (number of CpG per locus - *Peg3*: n=24; *Dlk1-Dio3:* n=27; *Igf2-H19:* n=16*;* PWS/AS: n=15) before and after swap (note: same data as in Fig. 4B for M FBS1 and M FBS5 iPSCs and as in Fig. 2A for M KSR5 iPSC before swap); On the bottom in A and B, chromatograms of *H19* and *Snrpn* genes are shown for M FBS1 and M FBS5 as well as M KSR3 and M KSR5, respectively, before and after medium swap (note: same chromatograms as in Fig. 4C for M FBS5). Source data are provided as Supplementary Data 2.

C. Representative flow cytometry plot for F FBS day12 (2) showing where gates were placed for collecting reprogramming [SSEA1+/THY1- (S+/T-) colored in green] and non-reprogramming [SSEA1-/THY1+ (S-/T+) colored in blue] intermediates. Mixed population is colored in orange.

D. RT-qPCR expression analyses of pluripotent markers (*Nanog* and *Esrrb*) normalised to the *Gapdh* gene in female cells collected during reprogramming (day12 and day24: T+/S- non-reprogramming intermediates; T-/S+ reprogramming intermediates) as well as in fully reprogrammed day50 F FBS2 and F FBS4 iPSCs; each bar represents data from only one biological replicate; Source data are provided as a Source Data file.

E. Graphs represent the average percentage ± SEM of 5mC and 5hmC per total cytosines in female and male cells sorted at day12 (T+/S-, n=1 for both biological sexes; female T-/S+, n=2; male T-/S+, n=1) and at day24 (female T-/S+, n=3;

male T-/S+, n=2), as well as in female and male day50 FBS-iPSCs (note: same data as in Fig. 5B, D for FBS-iPSCs, n=5 for both biological sexes); Source data are provided as a Source Data file.

F. Graph represents the mean percentage of methylation ± SEM considering both parental alleles of *Prickle1* gene in female and male cells sorted at day12 (T+/S-, n=1 for both biological sexes; female T-/S+, n=2; male T-/S+, n=1) and day24 (T-/S+, n=2 for both biological sexes) as well as in female and male day50 iPSCs (n=5 for both biological sexes); Source data are provided as Supplementary Data 2.

G. Allele-specific *H19* and *Snrpn* expression analysis assayed by Sanger sequencing. Chromatograms are shown for M FBS day24 clA and M FBS2.

# Supplementary Fig. 8

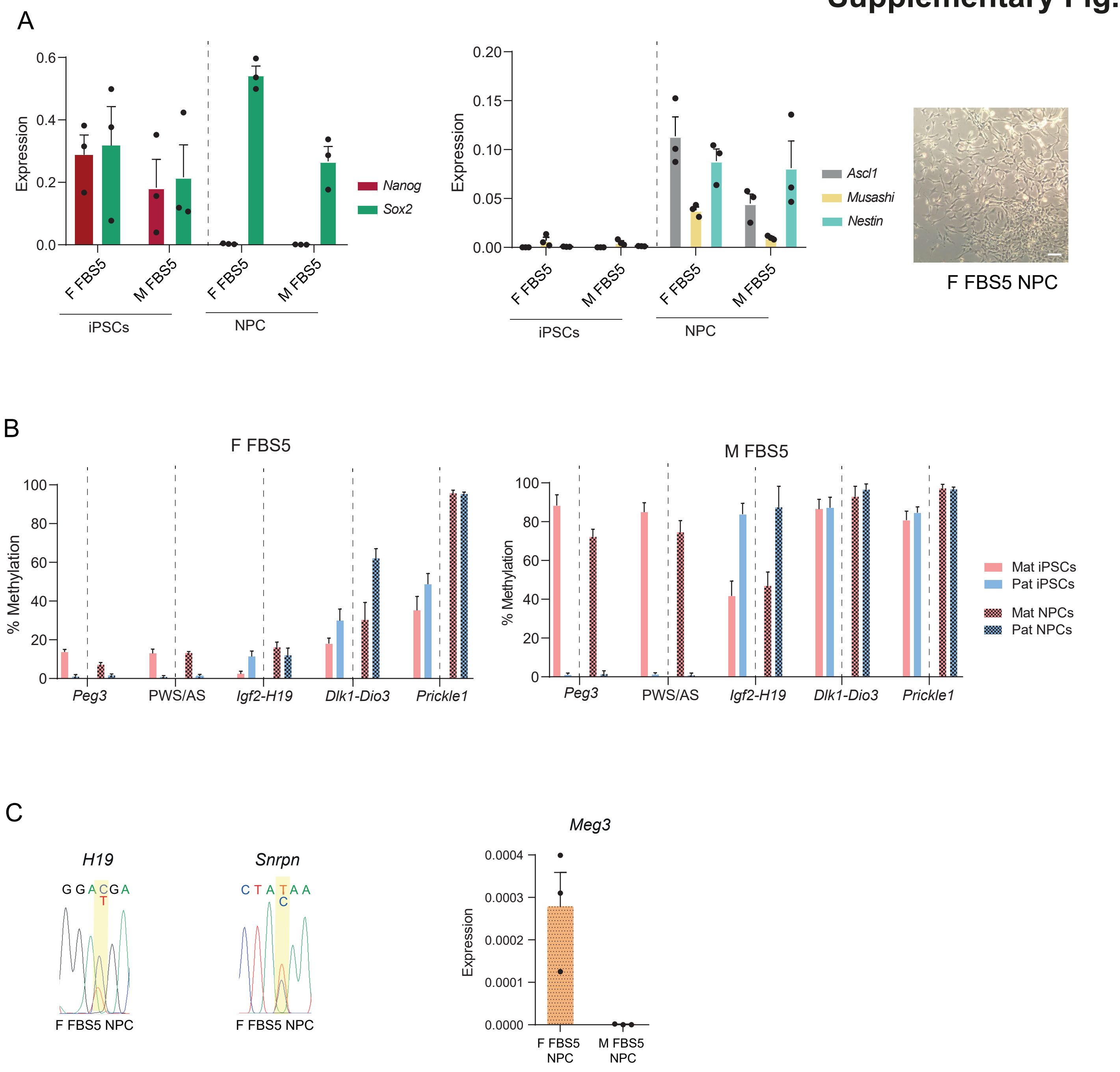

**Supplementary Figure 8 – Persistence of imprinting errors in iPSC-derived NPCs**

A. On the left, RT-qPCR expression analyses of pluripotent marker (*Nanog*), pluripotent and Neuronal Precursor Cell (NPC) marker (*Sox2*) and NPC-specific markers (*Ascl1*, *Musashi*, *Nestin*) normalised to the *Gapdh* housekeeping gene in F FBS5, M FBS5 and their correspondent NPCs (n=3 biological independent replicates); Graph represents the mean expression ± SEM in iPSCs and corresponding NPCs. On the right, a representative bright-field image of the F FBS5 NPCs. Scale bar corresponds to 100 µm. Source data are provided as a Source Data file.

B. Methylation analysis of *Peg3,* PWS/AS*, Igf2-H19, Dlk1-Dio3* imprinted regions and *Prickle1* (methylated control) in F FBS5, M FBS5 iPSCs and correspondent NPCs (note: same data as in Fig. 4B for F FBS5 and M FBS5 iPSC lines); Each graph represents the mean ± SD methylation levels measured at each CpG within different genomic regions per parental allele for each sample (number of CpG per locus - *Peg3*: n=24; PWS/AS: n=15; *Igf2-H19:* n=16*; Dlk1-Dio3:* n=27; *Prickle1*: n=10); Source data are provided as Supplementary Data 2.

C. Allele-specific *H19* and *Snrpn* expression analysis assayed by Sanger sequencing and RT-qPCR expression analysis of *Meg3* normalised to the *Gapdh* housekeeping gene in F FBS5 and M FBS5 NPCs (n=3 biological independent replicates). Graph represents the mean expression ± SEM in F FBS 5 and M FBS 5 NPCs. Source data are provided as a Source Data file.

# Supplementary Fig. 9

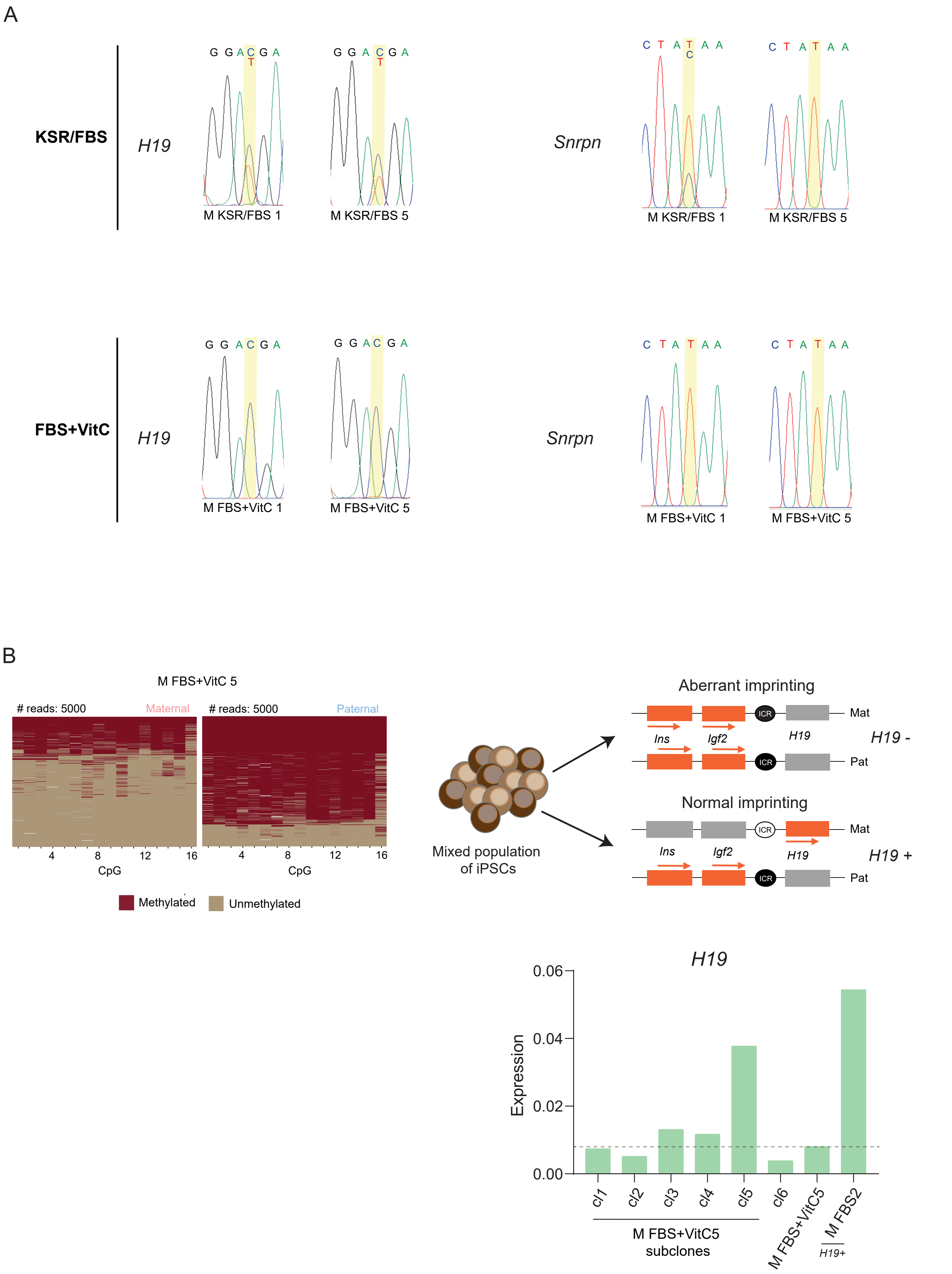

**Supplementary Figure 9 – Expression of imprinted genes in KSR/FBS- and FBS+VitC-iPSCs**

A. Allele-specific *H19* and *Snrpn* expression analysis assayed by Sanger sequencing in KSR/FBS- and FBS+VitC-iPSCs. Chromatograms for M KSR/FBS1 and M KSR/FBS5 (top) and for M FBS+VitC1 and M FBS+VitC5 (bottom) are shown.

B. On the left, plots displaying methylated and unmethylated CpGs for each CpG position (in columns) in all the individual reads (in rows) for both maternal and paternal alleles of *Igf2-H19* locus in M FBS+VitC5. On top right, the scheme represents M FBS+VitC5 iPSCs with a mixed population of cells with normal and abnormal imprinting as estimated by the plots presented on the left. The status of *Igf2-H19* ICR with normal and abnormal (hypermethylation) imprinting are also displayed (white circle – unmethylated ICR; black circle – methylated ICR; Mat – maternal allele; Pat – paternal allele; orange rectangles – expressed genes; grey rectangles – silenced genes; regions are not drawn to scale). On the bottom right, graph represents the RT-qPCR expression analysis of *H19* expression normalised with the *Gapdh* housekeeping gene in the subclones of M FBS+VitC5, original M FBS+VitC5 and M FBS2 (normal imprinting); dashed line represents the *Meg3* expression level of the parental M FBS+VitC5 iPSC line; each bar represents data from only one biological replicate. Source data are provided as a Source Data file.

FACS gating strategy

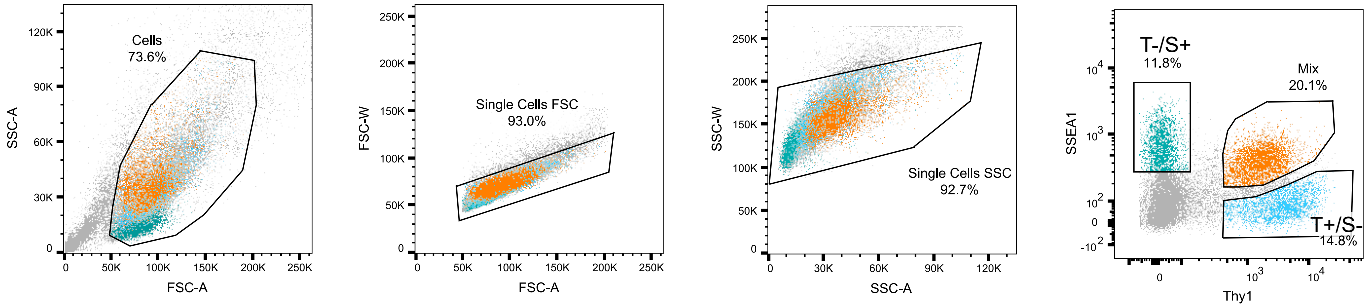


**Supplementary Figure 10 – FACS gating strategy**

Gating strategy used for FACS-sorting of THY1+/SSEA1- (T+/S-) non-reprogramming intermediates and THY1-/SSEA1+ (T-/S+) reprogramming intermediates. Cells were gated in an FSC and SSC intensity dot plot to eliminate debris. Doublets were excluded based on FCS-W, FSC-A and SSC-W, SSC-A. Then non-reprogramming and reprogramming intermediates populations were discriminated based on CD90.2/Thy1.2 and CD15/SSEA-1 expression (note: last plot on the right is the same data as in Supplementary Fig. 7C). FSC: Forward scatter, SSC: Side scatter.

| | Article | Stadtfeld et al., (2010) | Sun, et al., (2012) | Takikawa et al., (2013) | Yagi,et al., (2019) |
|---|---|---|---|---|---|
| Reprogramming | System | DOX-inducible, OKSM in *Col1a1* locus + *rtTA* in *Rosa26* locus | Retrovirus vsv-pseudotyped expressing individual human K,O,S,M | DOX-inducible, OSKM in *Col1a1* locus + *rtTA* in *Rosa26* locus | *PiggyBac* vector containing a DOX-inducible OSKM + *rtTA* |
| | Culture Conditions | 15% FBS | 20% KSR | 15% FBS | 15% FBS |
| iPSCs lines | Strain | ——— | Reciprocal crosses of C57BL/6J x Cast/Ei | (F)DBA/2 x (M)129 | (F)129X1/SvJ x (M) MSM/Ms |
| | Number | 6 | 8 | 12 | 5 (and 1 pre-iPSC) |
| | Gender | Not defined | Female and Male | Not defined | Male |
| Imprinting analyses | Imprinted clusters analysed | *Dlk1-Dio3* | PWS/AS, *Peg3*, *Kcqn1-Kcnq1ot1* , *Dlk1-Dio3*, *Igf2-H19*, *Mcts2-H13* | *Dlk1-Dio3*, *Igf2-H19, Rasgrf1*, *Mest/Peg1*, *Peg3*, *Sgce-Peg10*, PWS/AS, *Plagl1/Zac1* | Virtually all |
| | Methylation method | Bisulfite Pyrosequencing | Bisulfite Pyrosequencing | COBRA / Bisulfite sequencing | Bisulfite sequencing MethylC-seq |
| | Expression | Affymetrix mRNA expression microarray & RT-qPCR | Pyrosequencing | Allele-specific RT-PCR | RNA-seq |
| | Parental allele distinction | No | No | Yes, only for *Zim1* and *Snrpn* genes | Yes, large number of genes with SNPs |
| | Output | Hypermethylation of *Dlk1-Dio3* | Hypomethylation of PWS/AS, *Peg3* & *Kcnq1-Kcnq1ot1* (only female); *Dlk1-Dio3*, *Mcts2-H13* and *Igf2-H19* (male + female) | Hypomethylation of *Dlk1-Dio3*, *Igf2-H19, Rasgrf1*, *Peg3*, *Sgce-Peg10*, PWS/AS, *Plagl1/Zac1* Hypermethylation of *Mest/Peg1* | Hypermethylation of *Dlk1-Dio3* and *Igf2-H19* |

**Supplementary Table 1 –** Previous studies addressing imprinting defects in mouse iPSCs

Abbreviations: DOX - Doxycycline; OSKM – *Oct4*, *Sox2*, *Klf4*, *c-Myc*; FBS – Fetal Bovine Serum; KSR – Knockout Serum Replacement; F - Female; M – Male.

| | Region | Primer name | Primer sequences | PCR conditions |
|---|---|---|---|---|
| Pyrosequencing | *Line1-A* | L1-A_F | AGATTGAGGTATATAGGGAAGTAGGTT | Tm: 55C |
| | | L1-A_R-[Btn] | [biot]ATCCACTCACCAAAAATCTTAAAAT | |
| | | L1-A seq | GGTATATAGGGAAGTAGGTTA | |
| | *Line1-T* | L1-T_F | GGTTGGGGAGGAGGTTTAAGTTATA | Tm: 55C |
| | | L1-T_R-[Btn] | [biot]CTACCTATTCCAAAAACTATCAAATTCTCT | |
| | | L1-T seq | GGGAGGAGGTTTAAGTTATAGTA | |
| | *IAP* | IAP F | GAGGGTGGTTTTTTATTTTATGTGT | Tm: 58C |
| | | IAP R [Btn] | [biot]ATCACTCCCTAATTAACTACAACC | |
| | | IAP seq | TTTTTATTTTATGTGTTTTGTTTTT | |
| RT-qPCR | *Pou5f1* | mPou5f1 Fw | CCGGAAGAGAAAGCGAACTA | Tm: 60C |
| | | mPou5f1 Rv | CGCCGGTTACAGAACCATAC | |
| | *Nanog* | mNanog Fw | CCAGTCCCAAACAAAAGCTC | Tm: 60C |
| | | mNanog Rv | ATCTGCTGGAGGCTGAGGTA | |
| | *Esrrb* | mEsrbb3 Fw | TCTCATCTTGGGCATCGTGT | Tm: 60C |
| | | mEsrbb31 Rv | AGTTTCTTGTACCTGCGCAC | |
| | *Gapdh* | mGapdh Fw | AACTTTGGCATTGTGGAAGG | Tm: 60C |
| | | mGapdh Rv | ACACATTGGGGGTAGGAACA | |
| | *Meg3* | mMeg3 Fw | TTGCACATTTCCTGTGGGAC | Tm: 60C |
| | | mMeg3 Rv | AAGCACCATGAGCCACTAGG | |
| | *Dnmt1* | mDnmt1 Fw | ATCAGGTGTCAGAGCCCAAAG | Tm: 60C |
| | | mDnmt1 Rv | TGGTGGAATCCTTCCGATAAC | |
| | *Dnmt3a* | mDnmt3a Fw | AGACGTCTCCAACATGAGCC | Tm: 60C |
| | | mDnmt3a Rv | TTTCTCTTCTGGGTGCTGAAC | |
| | *Dnmt3b* | Dnmt3b Fw | CCCTTGAAGGACTACTTTGCC | Tm: 60C |
| | | Dnmt3b Rv | AGAGAACATGAGCACCATGC | |
| | *Dnmt3L* | Dnmt3L Fw | ATGGACAATCTGCTGCTGACTG | Tm: 60C |
| | | Dnmt3L Rv | CGCATAGCATTCTGGTAGTCTCTG | |
| | *Tet1* | mTet1 Fw | TTTGGTTCGTGAGCGTGTAG | Tm: 60C |
| | | mTet1 Rv | TGCAGGTACGCTTTTTGTTG | |
| | *Tet2* | mTet2 Fw | AACCTGGCTACTGTCATTGCTCCA | Tm: 60C |
| | | mTet2 Rv | AGATGTTCTGCTGGTCTCTGTGGGAA | |
| | *Tet3* | mTet3 Fw | TGCGATTGTGTCGAACAAATAGT | Tm: 60C |
| | | mTet3 Rv | TCCATACCGATCCTCCATGAG | |
| | *Zfp57* | mZfp57 Fw | CACAAATCCACAAAGCCGCAA | Tm: 60C |
| | | mZfp57 Rv | TGAACGGGGCCTATAACCTAAA | |
| | *Trim28* | mTrim28 Fw | CGCATGTATCAGGCATGAAG | Tm: 60C |
| | | mTrim28 Rv | CTTCCAGGAAAGACCTTGAAGA | |
| | *Dppa3* | mDppa3 Fw | GACCCAATGAAGGACCCTGAA | Tm: 60C |
| | | mDppa3 Rv | GCTTGACACCGGGGTTTAG | |
| | *Uhrf1* | mUhrf1 Fw | CCCAGGTGGTCCAGGTACAG | Tm: 60C |
| | | mUhrf1 Rv | CACGAGCACGGACATTCTTG | |
| | *Sox2* | mSox2 Fw | ATGCACCGCTACGACGTCA | Tm: 60C |
| | | mSox2 Rv | CTTTTTGCACCCCTCCCAATT | |
| | *Xist* | mXist Fw | GCTGGTTCGTCTATCTTGTGGG | Tm: 60C |
| | | mXist Rv | CAGAGTAGCGAGGACTTGAAGAG | |
| | *Mecp2* | mMecp2 Fw | TGACTTCACGGTAACTGGGAG | Tm: 60C |
| | | mMecp2 Rv | TTTCACCTGAACACCTTCTGATG | |
| | *Huwe1* | mHuwe1 Fw | GAGGGCGTAAACATACAGAGAAG | Tm: 60C |
| | | mHuwe1 Rv | CGCTGCTGTGTAAAGTGGC | |
| | *Jarid1c* | mJarid1c Fw | GAGGCCCAGACAAGAGTGAAA | Tm: 60C |
| | | mJarid1c Rv | TTGGGAATCTTTAAGGATGAGCC | |
| | *Ascl1* | mMash1 Fw | CCAACTACTCCAACGACTTGAACTC | Tm: 60C |
| | | mMash1 Rv | TCCTGCCATCCTGCTTCCAAAG | |
| | *Musashi* | mMsi1 Fw | CCGGAGTTACACAGGCCTTG | Tm: 60C |
| | | mMsi1 Rv | GGGATAGCTGTGAGCTCGGG | |
| | *Nestin* | mNestin Fw | CTCCTGTGACAGCCTTTCTGAAG | Tm: 60C |

| | | | | |
|---|---|---|---|---|
| | | mNestin Rv | AGGATAGGGAGCCTCAGACATAGG | |
| Genotyping | Yamanaka cassette | mPpary Fw | CAGCATCAAATGGCTCGGTA | Tm: 59C |
| | | mLenti Rv | GCACCATCCAAAGGTCAGTG | |
| | rtTA cassette (*Rosa26 locus*) | mRosa26 wt Fw | AAAGTCGCTCTGAGTTGTTAT | Tm: 59C |
| | | mRosa26 wt Rv | GGAGCGGGAGAAATGGAATG | |
| | | mRosa26 mut Rv | GCGAAGAGTTTGTCCTCAACC | |
| Allelic expression | *H19* | mH19 Fw | GCAATGCTGCCCCAGTAC | Tm: 61C |
| | | mH19 Rv | GACTAGGCGAGGGGAAGGC | |
| | *Snrpn* | mSnrpn Fw | CATTATGGCTCCTCCACCTG | Tm: 61C |
| | | mSnrpn Rv | GGGTCAAAAAGCTTGCAGGTAC | |
| | *Meg3* | mMeg3 Fw | CTTGCTGGCCCTGGAGAT | Tm: 61C |
| | | mMeg3 Rv | AACGTGTTGTGCGTGAAGTC | |

**Supplementary Table 2 –** Primers used for pyrosequencing, RT-qPCR, genotyping and allelic expression experiments

| | Locus | Primer name | Primer sequences | PCR conditions | Coordinates of CpGs analysed | Type of ICR |
|---|---|---|---|---|---|---|
| IMPLICON | ***Sox2*** | mSox2 Fw | CTACACGACGCTCTTCCGATCTTAGTTAGTTATATGGGTAGAGGATT | Tm: 55C | chr3:34649370-34649554 | N/A |
| | | mSox2 Rv | TGCTGAACCGCTCTTCCGATCTNNNNNNNNAATATTTCAAAAAACTAACAAAACC | | | |
| | ***Klf4*** | mKlf4 Fw | CTACACGACGCTCTTCCGATCTGGTTTTTTTTGTTAATATTGATGAT | Tm: 55C | chr4:55530381-55530595 | N/A |
| | | mKlf4 Rv | TGCTGAACCGCTCTTCCGATCTNNNNNNNNACAAATAAAAAACTCCTCTACAACC | | | |
| | ***Prickle1*** | mPrickle1 Fw | CTACACGACGCTCTTCCGATCTGTTGGTTAAGGAAATAGTTTTTTTT | Tm: 55C | chr15:93500632-93500729 | N/A |
| | | m Prickle1 Rv | TGCTGAACCGCTCTTCCGATCTNNNNNNNNCAACCTCTATAACCAATATACCCATAC | | | |
| | ***H13-Mcts2*** | mH13DMR Fw | CTACACGACGCTCTTCCGATCTAGTATTAGAATATTGGGGGATTTT | Tm: 57C | chr2:152686786-152686933 | Maternal |
| | | mH13DMR Rv | TGCTGAACCGCTCTTCCGATCTNNNNNNNNAATTAAAAAATAAACAACCACTTC | | | |
| | ***Gnas*** | mGnasDMR Fw | CTACACGACGCTCTTCCGATCTATTTTGGATTAAAGTTTAAAGTGTT | Tm: 54C | chr2:174295708-174295902 | Maternal |
| | | mGnasDMR Rv | TGCTGAACCGCTCTTCCGATCTNNNNNNNNACAATTATACACCATACAAATAAATATATT | | | |
| | ***Peg3*** | mPeg3DMR Fw | CTACACGACGCTCTTCCGATCTTTTTGTAGAGGATTTTGATAAGGAG | Tm: 60C | Chr7:6730141-6730468 | Maternal |
| | | mPeg3DMR Rv | TGCTGAACCGCTCTTCCGATCTNNNNNNNNCAACCTTATCAATTACCCTTAAAAA | | | |
| | **PWS/AS** | mSnrpnDMR Fw | CTACACGACGCTCTTCCGATCTAATTTGTGTGATGTTTGTAATTATTTGG | Tm: 60C | Chr7:60005043-60005284 | Maternal |
| | | mSnrpnDMR Rv | TGCTGAACCGCTCTTCCGATCTNNNNNNNNATAAAATACACTTTCACTACTAAAATCC | | | |
| | ***Igf2-H19*** | mH19DMR Fw | CTACACGACGCTCTTCCGATCTGTAAGGAGATTATGTTTTATTTTTGG | Tm: 56C | Chr7:142581761-142582087 | Paternal |
| | | mH19DMR Rv | TGCTGAACCGCTCTTCCGATCTNNNNNNNNCCTCATAAAACCCATAACTAT | | | |
| | ***Kcnq1-Kcnq1ot1*** | mKvDMR Fw | CTACACGACGCTCTTCCGATCTTAAGGTGAGTGGTTTAGGAT | Tm: 60C | Chr7:143295094-143295470 | Paternal |
| | | mKvDMR Rv | TGCTGAACCGCTCTTCCGATCTNNNNNNNNAATCCCCCACACCTAAATTC | | | |
| | ***Commd1-Zrsr1*** | mCommd1DMR Fw | CTACACGACGCTCTTCCGATCTTAGGAGTATTTTTATTGTAGGGGG | Tm: 60C | chr11:22971952-22972131 | Maternal |
| | | mCommd1DMR Rv | TGCTGAACCGCTCTTCCGATCTNNNNNNNNCTAAAACAATAATTATCTACCTTACC | | | |
| | ***Dlk1-Dio3*** | mIG-DMR Fw | CTACACGACGCTCTTCCGATCTGTGGTTTGTTATGGGTAAGTTT | Tm: 60C | Chr12:109528253-109528471 | Paternal |
| | | mIG-DMR Rv | TGCTGAACCGCTCTTCCGATCTNNNNNNNNCCCTTCCCTCACTCCAAAAATTAA | | | |
| | ***Mest/Peg1*** | mPeg1 DMR Fw | CTACACGACGCTCTTCCGATCTTTTTTGTTATTATGGGTTAAGGG | Tm: 54C | Chr6:30737609-30737809 | Maternal |
| | | mPeg1 DMR Rv | TGCTGAACCGCTCTTCCGATCTNNNNNNNNCCTCTTTCCTTATAAATCTCTAA | | | |
| | ***Plagl1/Zac1*** | mPlagl1 Fw | CTACACGACGCTCTTCCGATCTTTGTAGTTAGAGATGTAGTAAAAG | Tm: 54C | Chr10:13091188-13091317 | Maternal |
| | | mPlagl1 Rv | TGCTGAACCGCTCTTCCGATCTNNNNNNNNTTCCCCAAAAAAACACAAAT | | | |
| | ***Grb10*** | mGrb10 Fw | CTACACGACGCTCTTCCGATCTTTTTAAGGAGAAAAAAGGTT | Tm: 50C | Chr11:12025411-12025700 | Maternal |
| | | mGrb10 Rv | TGCTGAACCGCTCTTCCGATCTNNNNNNNNCCAAATAATAAACAACTCCT | | | |
| | ***Igf2r*** | mIgf2r Fw | CTACACGACGCTCTTCCGATCTAGGGTGAAAAGTTGTATAAG | Tm: 56C | Chr17:12742173-12742488 | Maternal |
| | | mIgf2r Rv | TGCTGAACCGCTCTTCCGATCTNNNNNNNNAATTTACACCCTCAAAATAC | | | |
| | ***Impact*** | mImpact Fw | CTACACGACGCTCTTCCGATCTGTGGATGAGGTGTATAATTT | Tm: 56C | Chr18:12972868-12973155 | Maternal |
| | | mImpact Rv | TGCTGAACCGCTCTTCCGATCTNNNNNNNNACAAAACAAAACTAAACCTAC | | | |

**Supplementary Table 3 –** Primers used for IMPLICON experiments
