# Supplementary material for: Imprinting fidelity in mouse iPSCs depends on sex of donor cell and medium formulation
Source: Nat Commun. 2022 Sep 16;13:5432. doi: 10.1038/s41467-022-33013-5 (PMC9481624; doi:10.1038/s41467-022-33013-5)
Supplement: Supplementary file 3 — Description of Additional Supplementary Files [file 41467_2022_33013_MOESM3_ESM.pdf]

Description of Additional Supplementary Files

Supplementary Data 1: Differentially expressed genes between female and male KSR-iPSCs. Differential expression analysis was determined using both EdgeR and intensity difference filter (p-value < 0.05 with multiple testing correction using Benjamini and Hochberg correction for both), with the intersection between the two lists giving the highly confident differentially expressed genes. Sheet 1: List of genes up or downregulated in female versus male KSR-iPSCs. Upregulated X-linked genes are highlighted; Sheet 2: Log2RPKM values for all the genes for all triplicates of female MEFs, F KSR2, F KSR4, M KSR3, M KSR5 iPSCs and TX 2i ESCs. Differentially expressed genes in female versus male iPSCs are highlighted in violet for upregulated X-linked genes, in pink for upregulated autosomal genes and purple for downregulated genes.

Supplementary Data 2: IMPLICON data. Average percentage of methylation levels and read counts at each CpG within each parental allele of imprinted or control genomic regions. Each row represents methylation levels or read counts for individual CpG analysed; the percentages of DNA methylation are colour-coded, lowest numbers in red, highest in green; in columns: exact CpG position in the genome (column A), imprinted region amplified (column B); average % of DNA methylation per parental allele in each sample and individual read counts per parental allele in each sample (several column depending on the sheet). Data of different IMPLICON experiments/type of samples are presented in different spreadsheets with the correspondent names (KSR-iPSCs, FBS-iPSCs; KSR/FBS-iPSCs, FBS+VitC-iPSCs, NPCs, reprogramming intermediates and swap experiments).

Supplementary Data 3: Normalised SNP read counts for a selection of imprinted genes in female MEFs, F KSR2, F KSR4, M KSR3 and M KSR5 iPSCs and TX 2i ESCs. Selection was based on the following criteria: (1) transcription from a single allele in MEFs (ratio: > 90%:10%), (2) Log2 RPKM > 1 expression in all iPSC replicates; (3) Normalised cumulative SNP-specific read counts > 5 in at least two of the three replicates of iPSCs.
