# Supplementary material for: Imprinting fidelity in mouse iPSCs depends on sex of donor cell and medium formulation
Source: Nat Commun. 2022 Sep 16;13:5432. doi: 10.1038/s41467-022-33013-5 (PMC9481624; doi:10.1038/s41467-022-33013-5)
Supplement: Supplementary file 7 — Reporting Summary [file 41467_2022_33013_MOESM7_ESM.pdf]

nature research

Corresponding author(s): Simão Teixeira da Rocha

Last updated by author(s): Jul 21, 2022

Reporting Summary

Nature Research wishes to improve the reproducibility of the work that we publish. This form provides structure for consistency and transparency in reporting. For further information on Nature Research policies, see our Editorial Policies and the Editorial Policy Checklist.

Statistics

For all statistical analyses, confirm that the following items are present in the figure legend, table legend, main text, or Methods section.

n/a	Confirmed	
☐	☒	The exact sample size (n) for each experimental group/condition, given as a discrete number and unit of measurement
☐	☒	A statement on whether measurements were taken from distinct samples or whether the same sample was measured repeatedly
☐	☒	The statistical test(s) used AND whether they are one- or two-sided<br>*Only common tests should be described solely by name; describe more complex techniques in the Methods section.*
☐	☒	A description of all covariates tested
☐	☒	A description of any assumptions or corrections, such as tests of normality and adjustment for multiple comparisons
☐	☒	A full description of the statistical parameters including central tendency (e.g. means) or other basic estimates (e.g. regression coefficient) AND variation (e.g. standard deviation) or associated estimates of uncertainty (e.g. confidence intervals)
☐	☒	For null hypothesis testing, the test statistic (e.g. F, t, r) with confidence intervals, effect sizes, degrees of freedom and P value noted<br>*Give P values as exact values whenever suitable.*
☒	☐	For Bayesian analysis, information on the choice of priors and Markov chain Monte Carlo settings
☒	☐	For hierarchical and complex designs, identification of the appropriate level for tests and full reporting of outcomes
☐	☒	Estimates of effect sizes (e.g. Cohen's d, Pearson's r), indicating how they were calculated

Our web collection on statistics for biologists contains articles on many of the points above.

Software and code

Policy information about availability of computer code

Data collection	H&E images were taken by a pathologist blinded to experimental groups using a Leica DM2500 microscope coupled to a Leica MC170 HD microscope camera. Fluorescent microscopy images were taken using Zeiss Axio Observer (Carl Zeiss MicroImaging) equipped with an Axiocam CCD camera using 63×/1.4 oil objective (Plan-Apochromat) with the filter sets FS43HE and FS49. All RT-qPCR data were collected on a ViiA-7 real-time thermal cycler (Applied Biosystems). RNA-seq datasets were acquired on an Illumina Hiseq 2000 platform, while IMPLICON datasets were acquired on an Illumina MiSeq platform and both data processed using a standard Illumina base-calling pipeline. 5mC and 5hmC measurements were collected on a LTQ Orbitrap Velos mass spectrometer (Thermo Scientific) fitted with a nanoelectrospray ion-source (Proxeon, Odense, Denmark). Pyrosequecing data was obtained using the PyroMark Q48 (Qiagen) device. Sanger sequencing was outsourced to STABVIDA and run on an ABI 3730 xl sequencer. FACS experiments to collect reprogramming and non-reprogramming intermediates were obtained using a BD FACSAria IIu sorter.

Data analysis	All data analyses in this study are extensively described in the Methods section of the paper. For imaging analysis, digital images were processed using FIJI platform (ImageJ v.2.1.0/1.53q; https://fiji.sc/). Downstream analyses of RT-qPCR, 5mC/5hmC measurements and pyrosequecing were performed on Microsoft Excel spreadsheet software (v2206 Build 16. 0. 15330. 20144) followed by statistical analysis on Graphpad Prism v8.0.1 program. Sanger sequencing data were visualized and analysed on a Chromas version 2.6.2 software. FACS experiments to collect reprogramming and non-reprogramming intermediates were analysed using FlowJo software v10.7.2. Pyrosequencing data was analysed using Q48 Autoprep (v2.4.2 Build 3), PyroMark Autoprep Q48 Software (v4.2.1) and Firmware (v4.03) and OS version (v2.0.0). For IMPLICON analysis, we followed the step-by-step guide of data processing analysis in https://github.com/FelixKrueger/IMPLICON as previously published (Klobucar et al.2020). Extensive details are given in the Methods section. Raw sequence reads were then trimmed to remove both poor quality calls and adapters using Trim Galore v0.5.0. Alignments were carried out with Bismark v0.20.0. Reads were split allele-specifically with SNPsplit package v0.3.4, https://github.com/FelixKrueger/SNPsplit).<br>RNAseq raw FastQ data were trimmed with Trim Galore (version 0.6.1, default parameters) and mapped to the mouse GRCm38 genome assembly using Hisat2 version 2.1.0. Alignments were mapped to both CAST_EiJ and C57BL/6 (GRCm38) genomes and aligned read files (bam) were imported to the Seqmonk software v1.47 for downstream analysis. EdgeR v3.26.7 was used to determine the list of differentially expressed genes between female and male KSR-iPSCs. Detailed information on RNAseq analysis is given in the Methods section.

For manuscripts utilizing custom algorithms or software that are central to the research but not yet described in published literature, software must be made available to editors and reviewers. We strongly encourage code deposition in a community repository (e.g. GitHub). See the Nature Research guidelines for submitting code & software for further information.

Data

Policy information about availability of data

All manuscripts must include a data availability statement. This statement should provide the following information, where applicable:

- Accession codes, unique identifiers, or web links for publicly available datasets
- A list of figures that have associated raw data
- A description of any restrictions on data availability

Source data are provided with this paper and all the sequencing datasets produced in this study are available at Gene Expression Omnibus GSE148067 (https://www.ncbi.nlm.nih.gov/geo/query/acc.cgi?acc=GSE148067).

Field-specific reporting

Please select the one below that is the best fit for your research. If you are not sure, read the appropriate sections before making your selection.

☒ Life sciences ☐ Behavioural & social sciences ☐ Ecological, evolutionary & environmental sciences

For a reference copy of the document with all sections, see nature.com/documents/nr-reporting-summary-flat.pdf

Life sciences study design

All studies must disclose on these points even when the disclosure is negative.

Sample size	The sample size were chosen to ensure the findings could be reproduced by performing multiple independent experiments (usually n>= 3). We also generate 5-to-6 iPSCs for each experimental group. Sample size was chosen according to prior knowledge and practices in the field.
Data exclusions	For the IMPLICON data deposited in GEO under the accession number GSE148067, specific reads mapped to the following murine (mm10) genomic coordinates were excluded for consideration in this article for one of the following reasons: (1) regions that fail to reach the coverage threshold for the two parental alleles in a given sample (> 40 reads); (2) regions sequenced twice for which only the run with more reads was considered; (3) regions out of the scope of this article. This information is clearly stated in the Methods section.
Replication	The majority of the experiments presented were replicated. If not, two independent methods (e.g., IMPLICON and relative allele expression by RT-PCR followed by Sanger sequencing) were applied. All the key experiments presented in this manuscript were reproduced using 5-6 iPSC lines generated per experimental condition.
Randomization	For the IMPLICON experiments, samples were randomized for the iTAG primers addition prior to Illumina sequencing. When it was not possible to randomize, covariates were controlled using appropriate positive/negative controls (e.g., using samples with normal imprinting; blanks for PCR experiments).
Blinding	Teratoma analysis by a pathologist, IMPLICON and RNAseq analyses by a bioinformatician and pyrosequencing and 5mC/5hmC measurements were executed without prior knowledge of the identity of each sample and data collection was therefore blinded to investigators. RT-qPCR, IF or Sanger sequencing data were not blinded as, for practical reasons for the accomplishment of the experiment, it was necessary for the operator not to be blinded.

Reporting for specific materials, systems and methods

We require information from authors about some types of materials, experimental systems and methods used in many studies. Here, indicate whether each material, system or method listed is relevant to your study. If you are not sure if a list item applies to your research, read the appropriate section before selecting a response.

Materials & experimental systems

n/a	Involved in the study
	☒ Antibodies
	☒ Eukaryotic cell lines
☒	Palaeontology and archaeology
	☒ Animals and other organisms
☒	Human research participants
☒	Clinical data
☒	Dual use research of concern

Methods

n/a	Involved in the study
☒	ChIP-seq
	☒ Flow cytometry
☒	MRI-based neuroimaging

Antibodies

Antibodies used

Antibodies have been reported in the immunofluorescence and FACS sections of the Methods:
SSEA1 monoclonal clone MC-480 - Cat# MAB4301 – Merck Millipore
OCT4 monoclonal clone 7F9.2 - Cat# MAB4419 – Merck Millipore
StemAb™ Anti Mouse Nanog Antibody Cat# RCAB002P-F REPROCELL
PE anti-mouse CD90.2/Thy1.2- clone 30-H12 - Cat# 105307 – Biolegend
Brilliant Violet 421 anti-mouse CD15/SSEA-1, MC-480 clone - Cat# 125613 - Biolegend
Cy™3 AffiniPure F(ab')$_2$ Fragment Goat Anti-Mouse IgG (H+L) – Cat#115-166-003 Jackson ImmunoResearch Laboratories Inc.

Validation

In all circumstances, antibodies behaved as expected. Antibodies used for IF produced the expected staining patterns (nuclear for OCT4 and NANOG and membrane-bound for SSEA1). Antibodies used for FACS sorting isolated the expected cell populations as validated by RT-qPCR.

All antibodies used are commercial and validation data are available from supplier websites, as follows:

SSEA1 monoclonal clone MC-480 - Cat# MAB4301 – Merck Millipore: https://www.merckmillipore.com/PT/en/product/Anti-SSEA1-Antibody-clone-MC-480-Cy3-conjugate,MM_NF-MAB4301C3?ReferrerURL=https%3A%2F%2Fsearch.yahoo.com%2F&bd=1

OCT4 monoclonal clone 7F9.2 - Cat# MAB4419 – Merck Millipore:
https://www.merckmillipore.com/PT/en/product/Anti-OCT-4-POU5F1-Antibody-clone-7F9.2,MM_NF-MAB4419?ReferrerURL=https%3A%2F%2Fsearch.yahoo.com%2F&bd=1

StemAb™ Anti Mouse Nanog Antibody Cat# RCAB002P-F REPROCELL:
https://www.reprocell.com/product-catalog/antibodies-and-staining-kits/stemab-anti-mouse-nanog-antibody

PE anti-mouse CD90.2/Thy1.2- clone- 30-H12 - Cat# 105307 – Biolegend:
https://www.biolegend.com/en-us/products/pe-anti-mouse-cd90-2-thy1-2-antibody-106?GroupID=BLG1941

Brilliant Violet 421 anti-mouse CD15/SSEA-1, MC-480 clone - Cat# 125613 - Biolegend.
This antibody has been recently discontinued by the supplier. The same antibody is now available from a different supplier: https://www.fishersci.at/shop/products/anti-ssea-1-brilliant-violet-421-clone-mc480-bd/15812169

Cy™3 AffiniPure F(ab')$_2$ Fragment Goat Anti-Mouse IgG (H+L) – Cat#115-166-003 Jackson ImmunoResearch Laboratories Inc:
https://www.jacksonimmuno.com/catalog/products/115-166-003

Eukaryotic cell lines

Policy information about cell lines

Cell line source(s)	TX1072 ESCs (published in Schulz et al., 2014; from the Institute Curie); JM8.F6 ESCs (published in Pettitt et al., 2009, kind gift from Manuel Serrano, IRB, Barcelona).
Authentication	TX1072 and JM8.F6 ESC lines have been used in multiple publications from different labs.
Mycoplasma contamination	The cells were screened for mycoplasma contamination on a regular basis using qPCR Mycoplasma Test (Mycoplasmacheck, Eurofins Genomics) and always found to be negative.
Commonly misidentified lines (See ICLAC register)	No cell lines used in this paper listed in the database of misidentified cell lines maintained by ICLAC.

Animals and other organisms

Policy information about studies involving animals; ARRIVE guidelines recommended for reporting animal research

Laboratory animals	Mouse Strains:<br><br>- “Reprogrammable'' transgenic i4F-BL6 (Mus musculus, C57BL/6J; Abad et al., 2013): 8-week-old females.<br>- CAST (Mus musculus castaneus, CAST/EiJ, the Jackson Laboratory): 12-week-old males.<br>- NSG mice (Mus muluscus, NOD.Cg- Prkdcscid Il2rgtm1Wjl/SzJ, the Jackson Laboratory; Shultz et al., 2007): 3-month-old males.<br><br>All mice colonies were maintained at the Instituto de Medicina Molecular João Lobo Antunes (iMM JLA) Rodent facility.
Wild animals	No wild animals were used in the study.
Field-collected samples	No field collected samples were used in the study.
Ethics oversight	Animal Ethics Committee of IMM JLA; Portuguese competent authority – Direcção Geral de Alimentação e Veterinária.

Note that full information on the approval of the study protocol must also be provided in the manuscript.

Flow Cytometry

Plots

Confirm that:

- [x] The axis labels state the marker and fluorochrome used (e.g. CD4-FITC).
- [x] The axis scales are clearly visible. Include numbers along axes only for bottom left plot of group (a 'group' is an analysis of identical markers).
- [x] All plots are contour plots with outliers or pseudocolor plots.
- [x] A numerical value for number of cells or percentage (with statistics) is provided.

Methodology

Sample preparation	Female and male MEFs were reprogrammed under FBS conditions and reprogramming intermediates (SSEA1+/THY1-) and non-reprogramming intermediates (SSEA1-/THY1+) were sorted at day12 and day24 of reprogramming. Incubation with 1:100 of the antibody PE anti-mouse CD90.2/Thy1.2 (Biolegend, Catalog N. 105307) and 1:200 of the antibody Brilliant Violet 421 anti-mouse CD15/SSEA-1 (Biolegend, Catalog N. 125613) was performed at 4ºC for 30 min and then stained cells were resuspended in FACS Buffer (PBS+2%FBS v/v) and transferred to cytometry tubes to be sorted.
Instrument	BD FACSAria IIu
Software	Flowjo v10.7.2
Cell population abundance	Reprogramming intermediates (SSEA1+/THY1-) and non-reprogramming intermediates (SSEA1-/THY1+) were sorted with a purity ≥98%; each sorted population corresponded to ~5-10% of the total number of cells.
Gating strategy	Doublets and dead cells were excluded by FSC and SSC.

- [x] Tick this box to confirm that a figure exemplifying the gating strategy is provided in the Supplementary Information.
